# Homozygous *Smpd1* deficiency aggravates brain ischemia/ reperfusion injury by mechanisms involving polymorphonuclear neutrophils, whereas heterozygous *Smpd1* deficiency protects against mild focal cerebral ischemia

**DOI:** 10.1007/s00395-020-00823-x

**Published:** 2020-10-14

**Authors:** Nina Hagemann, Ayan Mohamud Yusuf, Carlotta Martiny, Xiaoni Zhang, Christoph Kleinschnitz, Matthias Gunzer, Richard Kolesnick, Erich Gulbins, Dirk M. Hermann

**Affiliations:** 1 Department of Neurology, University Hospital Essen, University of Duisburg-Essen, 45147 Essen, Germany; 2 Institute of Experimental Immunology and Imaging, University Hospital Essen, University of Duisburg-Essen, 45147 Essen, Germany; 3 Memorial Sloan Kettering Cancer Center, New York City, NY USA; 4 Department of Molecular Biology, University Hospital Essen, University of Duisburg-Essen, 45147 Essen, Germany

**Keywords:** Ceramide-rich membrane microdomain, Focal cerebral ischemia, Middle cerebral artery occlusion, Inflammation, Intercellular adhesion molecule-1, Leukocyte, Lipid raft, Sphingolipid

## Abstract

By cleaving sphingomyelin into ceramide, which is an essential component of plasma membrane microdomains, acid sphingomyelinase (Asm) pivotally controls cell signaling. To define how the activation of the Asm/ceramide pathway, which occurs within seconds to minutes upon stress stimuli, influences brain ischemia/reperfusion (I/R) injury, we exposed male and female wildtype mice carrying both alleles of Asm’s gene *sphingomyelinase phosphodiesterase-1* (*Smpd1*^+*/*+^), heterozygously Asm-deficient mice (*Smpd1*^+/−^) and homozygously Asm-deficient mice (*Smpd1*^*−/−*^) of different age (8, 12 or 16 weeks) to 30, 60 or 90 min intraluminal middle cerebral artery occlusion (MCAO). For studying the contribution of brain-invading polymorphonuclear neutrophils (PMN) to I/R injury, PMNs were depleted by delivery of a PMN-specific Ly6G antibody. In male and female mice exposed to 30 min, but not 60 or 90 min MCAO, homozygous *Smpd1*^*−/−*^ consistently increased I/R injury, blood–brain barrier permeability and brain leukocyte and PMN infiltration, whereas heterozygous *Smpd1*^+/−^ reduced I/R injury. Increased abundance of the intercellular leukocyte adhesion molecule ICAM-1 was noted on cerebral microvessels of *Smpd1*^*−/−*^ mice. PMN depletion by anti-Ly6G delivery prevented the exacerbation of I/R injury in *Smpd1*^*−/−*^ compared with wildtype mice and reduced brain leukocyte infiltrates. Our results show that Asm tempers leukocyte entry into the reperfused ischemic brain, thereby attenuating I/R injury.

**Electronic supplementary material:**

The online version of this article (10.1007/s00395-020-00823-x) contains supplementary material, which is available to authorized users.

## Introduction

Acid sphingomyelinase (Asm), which catalyzes the hydrolysis of sphingomyelin to ceramide, is a key enzyme in sphingolipid metabolism. Localized in lysosomes, Asm is translocated to the plasma membrane within a few seconds to minutes upon stress stimuli [1–3]. In plasma membranes, ceramide clusters into membrane platforms that critically control the activity of signal pathways modifying cell survival and death [1, 2, 4, 5]. Several stress stimuli activating Asm and inducing ceramide-rich membrane domains were identified, including γ-irradiation, UV light exposure, ischemia/reperfusion (I/R), bacterial infection and chemotherapy [6–11]. Asm-dependent ceramide signaling was shown to control T cell CD3 signaling [12, 13], regulate CD4^+^ T cell activation and proliferation [12, 13], promote macrophage cytokine release [14, 15] and augment macrophage phagocytosis and apoptosis [12, 13]. Importantly, the pharmacological inhibition or genetic depletion of Asm in mice conferred protection against cystic fibrosis, lung infection and sepsis [15, 16], whereas Asm-dependent and -independent ceramide formation was found to be required for radiation-induced tumor apoptosis [10, 17]. Hence, the deactivation of Asm represents a potent strategy for the prevention of cell injury in a wide range of pathologies.

In the brain, Asm has been recognized as plasticity-promoting therapeutic target. In a model of stress-induced depression, the antidepressants amitriptyline and fluoxetine, which are diverse in their chemical structure and neurotransmitter mode of action, inhibited cerebral Asm activity, restored neuronal proliferation and differentiation in the hippocampus, which under conditions of depression is compromised, and reversed depressive-like behaviors [18]. In *sphingomyelinase phosphodiesterase-1* (*Smpd1*)^*−/−*^ mice lacking Asm, both antidepressants did not increase neuronal proliferation and differentiation and did not reverse depressive-like behaviors [18]. Hence, inhibition of Asm is indispensable for the mood-stabilizing action of antidepressive drugs [19]. In humans, the complete loss of Asm activity in subjects exhibiting autosomally recessive *SMPD1* mutations results in Niemann-Pick disease type-A, characterized by neurodegeneration of the cerebral and cerebellar cortex, basal ganglia, brain stem and spinal cord with ataxia, dysarthria and dysphagia [20]. The *Smpd1*^*−/−*^ mouse develops a very similar clinical and histopathological phenotype characterized by progressive neurodegeneration and ataxia starting at the age of 4–6 months followed by animal death at ~ 8 months [21]. This suggests that a basal Asm activity is required for maintaining brain integrity and function.

So far, limited information exists how the deactivation of the Asm/ceramide pathway influences brain injury after focal cerebral ischemia/ reperfusion (I/R). A single study evaluating effects of *Smpd1*^*−/−*^ after transient intraluminal middle cerebral artery occlusion (MCAO) in mice found that Asm deficiency protected against I/R injury and reduced pro-inflammatory cytokine production [22]. In vitro, primary cerebral cortical neurons obtained from *Smpd1*^*−/−*^ mice exhibited decreased vulnerability to glutamate excitotoxicity, which was associated with decreased levels of intracellular calcium and free oxygen radicals [22]. To elucidate the role of the Asm/ceramide pathway in brain I/R injury, we exposed male or female *Smpd1*^+*/*+^, *Smpd1*^+/−^ and *Smpd1*^*−/−*^ mice of different age (8, 12 or 16 weeks) to intraluminal MCAO of different duration (30, 60 or 90 min). Based on the observation that *Smpd1*^*−/−*^ mice exhibited exacerbated brain infarcts associated with increased brain leukocyte and polymorphonuclear neutrophil (PMN) infiltrates, whereas *Smpd1*^+/−^ revealed reduced brain infarcts, we subsequently depleted PMNs in *Smpd1*^+*/*+^ and *Smpd1*^*−/−*^ mice using anti-lymphocyte antigen-6 (locus G; Ly6G) antibody, evaluating the role of PMNs for I/R injury.

## Materials and methods

### Animal groups, randomization and blinding

Experiments were performed with local government approval (Landesamt für Natur, Umwelt und Verbraucherschutz, Northrhine Westphalia) in accordance to E.U. guidelines (Directive 2010/63/EU) for the care and use of laboratory animals and reported based on Animal Research: Reporting In Vivo Experiments (ARRIVE) guidelines. Eight, 12- or 16-week-old male or female *Smpd1*^+/+^, *Smpd1*^+/−^, and *Smpd1*^−/−^ animals were bred from *Smpd1*^+/−^ mice on a C57BL/6 J background. Genotyping of ear tissue was performed as described before [21]. Experiments involving treatments were strictly randomized. The experimenter performing the surgeries and histochemical studies (N.H.) was blinded by another researcher (A.M.Y.) preparing the treatment solutions and blinding the genotypes, which received dummy names (A, B, C, D) and were decoded after termination of the study. For PMN depletion experiments, mice were injected with 200 μg of an isotype anti-mouse control antibody (Clone 2A3; BioXcell, Lebanon, NH, U.S.A.) or 200 µg of anti-mouse Ly6G (Clone 1A8; BioXcell) 24 h before and 24 h after MCAO [23]. Animals were kept in a regular 12 h:12 h light/dark cycle in groups of five animals/cage with free access to food and water. The data that support the findings of this study are available from the corresponding author upon reasonable request. 3D light sheet microscopy studies using FITC albumin gelatin as microvascular tracer [24], which were conducted in the preparation of this study, revealed that microvascular network characteristics in the brain, that is, microvascular length density, branching point density, mean branch length and tortuosity, did not differ between *Smpd1*^+/+^, *Smpd1*^+/−^ and *Smpd1*^−/−^ mice (Suppl. Figure 1).

### Induction of focal cerebral ischemia

For induction of focal cerebral ischemia, animals were anesthetized with 1.5% isoflurane (30% O_2_, 70% N_2_O). Rectal temperature was maintained between 36.5 °C and 37.0 °C with a feedback-controlled heating system. During the experiments, LDF was monitored with a flexible 0.5 mm fiber-optic probe (Perimed, Rommerskirchen, Germany) attached to the intact skull overlying the middle cerebral artery territory (2 mm posterior, 6 mm lateral from bregma). LDF changes were measured during MCAO and up to 15 min after reperfusion onset. Focal cerebral ischemia was induced with an intraluminal filament technique, as previously described [25]. Briefly, a midline neck incision was made, and the left common and external carotid arteries were isolated and ligated. A microvascular clip (FE691; Aesculap, Tuttlingen, Germany) was temporarily placed on the internal carotid artery. An 7–0 nylon monofilament (Doccol, Sharon, MA, U.S.A.) coated with silicone (diameter 200–210 μm) was introduced through a small incision into the common carotid artery and advanced 9 mm distal to the carotid bifurcation for MCAO. Thirty, 60 or 90 min after MCAO, reperfusion was initiated by withdrawal of the monofilament. After the surgery, wounds were carefully sutured, anesthesia was discontinued, and animals were allowed to recover. Analgesia was ensured by subcutaneous injection of 0.1 mg/kg buprenorphine (Temgesic^®^; Essex Pharma, Munich, Germany) before surgery and subcutaneous injection of 4 mg/kg Carprofen (Rimadyl^®^; Pfizer, New York, NY, U.S.A.) directly after MCAO and thereafter daily at 24-h-intervals. Mice were transcardially perfused with ice-cold 0.1 M phosphate-buffered saline (PBS) 24 h or 72 h post-MCAO. Brains were removed and fresh-frozen on dry ice. For histochemistry and immunohistochemistry, brains were cut on a cryostat into 20 µm coronal brain sections. In addition, tissue samples were taken from the middle cerebral artery territory of both hemispheres for Asm activity assays, Western blots, and reverse transcriptase-quantitative polymerase chain reaction (RT-qPCR) studies.

### Sphingomyelinase activity assay

Tissue samples obtained from the reperfused ischemic middle cerebral artery territory and homologous contralateral brain tissue were lysed in 250 mM sodium acetate buffer (pH 5.0) containing 1% NP-40 detergent (Fluka BioChemika, Morristown, NJ, U.S.A.). Cellular membrane integrity was disrupted with a sonicator. After centrifugation for 5 min at 300 g at 4 °C, supernatants were collected. Lysates were adjusted to a specific protein concentration and incubated with 100 pmol BODIPY-labeled sphingomyelin (Thermo Fisher Scientific) in 250 mM sodium acetate (pH 5.0) and 0.1% NP-40 for 1 h at 37 °C. Chloroform:methanol (2:1, v/v) was added, samples were vortexed and centrifuged for 5 min at 15,000 g to achieve a phase separation. The lower phase was collected and concentrated in a vacuum centrifuge (SPC111V, Thermo Fisher Scientific) for 45 min at 37 °C. Lipids were dissolved in 20 µl chloroform:methanol (2:1, v/v) and spotted onto thin layer chromatography (TLC) plates (Macherey Nagel, Düren, Germany). The TLC run was performed with chloroform:methanol (80:20, v/v). TLC plates were analyzed with a Typhoon FLA 9500 scanner (GE Healthcare Life Sciences) and lipid spots were quantified with ImageQuant (GE Healthcare Life Sciences).

### Brain infarct analysis

20 µm cryostat sections were collected at the rostrocaudal level of the striatum in all mice and stained with cresyl violet. The bregma level represents the center of the middle cerebral artery territory. Brain infarcts have their largest extension at this level and brain infarcts are most reproducible here [26, 27]. On the cresyl violet-stained brain sections, the border between infarcted and non-infarcted tissue was outlined using Image J (National Institutes of Health [NIH], Bethesda, MD, U.S.A.). Infarct area was determined by subtracting the area of the non-lesioned ipsilateral hemisphere from the area of the contralateral hemisphere [25]. In additional cohorts, cryostat sections were collected at millimeter intervals across the forebrain, which were stained with cresyl violet. On these sections, infarct areas were obtained at all forebrain levels, and infarct volume was calculated by integrating infarct areas across the forebrain [25]. Brain swelling was calculated as area difference of the ipsilateral and the contralateral hemisphere and expressed as percent increase in comparison to the contralateral hemisphere [25].

### Terminal deoxynucleotidyl transferase-mediated dUTP nick end labeling (TUNEL)

Adjacent brain sections at the bregma level were fixed with 4% paraformaldehyde (PFA) in 0.1 M PBS. Using a commercially available kit (In situ Cell Death Detection Kit; Roche, Basle, Switzerland), DNA-fragmented, that is, irreversibly injured cells were detected in these sections by terminal deoxynucleotidyl transferase dUTP nick end labeling (TUNEL) according to the manufacturer’s protocol. Sections were evaluated using an inverted microscope (Axio Observer.Z1; Carl Zeiss, Oberkochen, Germany) by counting the density of TUNEL + cells in a region of interest (ROI) measuring 1500 × 1500 µm in the dorsolateral reperfused ischemic striatum [25]. A schematic drawing demonstrating the precise location of this ROI is presented in Suppl. Figure 2.

### Immunohistochemistry

Adjacent sections from the bregma level were fixed with 4% PFA in 0.1 M PBS and incubated in 10% normal goat serum and 1% bovine serum albumin (BSA). Sections were incubated overnight at 4 °C in rat anti-CD45 (30-F11; BD Biosciences, Franklin Lakes, NJ, U.S.A.), rat anti-Ly6G (1A8; BD Biosciences), biotinylated goat anti-IgG (sc-2039; Santa Cruz, Heidelberg, Germany), goat anti-intercellular adhesion molecule-1 (ICAM-1; AF796; R&D Systems, Minneapolis, MN, U.S.A.) or rat anti-CD31 (MEC 13.3; BD Biosciences) antibody. Primary antibodies were detected with appropriate fluorescent or biotinylated secondary antibodies. Biotinylated antibodies were revealed by 3,3′-diaminobenzidine (DAB) staining using an avidin–biotin complex (ABC) peroxidase kit (Vectastain Elite Kit Standard; Vector Laboratories, Burlingame, CA, U.S.A.). Sections were evaluated using an inverted microscope (Axio Observer.Z1; Carl Zeiss, Oberkochen, Germany) by counting the density of labeled cells (CD45, Ly6G) or area covered by immunofluorescence signal (ICAM-1, ICAM-1/CD31) in a region of interest (ROI) measuring 1500 × 1500 µm in the dorsolateral reperfused ischemic striatum and contralateral striatum [25]. IgG extravasation was examined in the same ROI by densitometry [28]. A schematic drawing demonstrating the precise location of this ROI is given in Suppl. Figure 2.

### Western blot analysis

Tissue samples obtained from the reperfused ischemic middle cerebral artery territory and homologous contralateral brain tissue were lysed in NP40 lysis buffer (50 mM Tris/HCl pH 7.5, 150 mM NaCl, 0.5% NP-40, 2 mM EDTA) containing protease inhibitor cocktail (Roche, Mannheim, Germany). Equal protein amounts (20 µg) from individual animals were resolved by sodium dodecyl sulfate–polyacrylamide gel electrophoresis (SDS-PAGE) and transferred to nitrocellulose membranes (GE Healthcare Life Science, Little Charfont, U.K.). Non-specific binding was blocked for 1 h at room temperature with 5% skim milk powder (Sigma-Aldrich, Deisenhofen, Germany) dissolved in 0.1% Tween in 0.1 M Tris-buffered saline (TBS-T). Membranes were incubated overnight at 4 °C in goat anti-ICAM-1 (AF796; R&D Systems, Minneapolis, MN, U.S.A.) or rabbit anti-β-actin (4967; Cell Signaling Technology, Danvers, MA, U.S.A.) antibody diluted in TBS-T, followed by incubation for 1 h at room temperature in HRP-conjugated secondary antibody (Santa Cruz Biotechnology) diluted in TBS-T. Signals were detected by enhanced chemiluminescence using prime Western blotting detection reagent (GE Healthcare Life Science). ICAM-1 abundance was normalized to β-actin abundance.

### Reverse transcriptase-quantitative polymerase chain reaction (RT-qPCR)

Tissue samples obtained from the reperfused ischemic middle cerebral artery territory and homologous contralateral brain tissue were homogenized in 250 µl Trizol (Invitrogen, Carlsbad, CA, U.S.A.) using a glass-teflon homogenizer. Chloroform was added, and phases were separated by centrifugation. RNA was precipitated by adding 2-propanol. The RNA pellet was washed with 75% ethanol and dissolved in RNase-free water after drying. Reverse transcription of mRNA to cDNA was performed using Random Hexamer Primers (SuperScript II Reverse Transcriptase; Life Technologies, Carlsbad, CA, U.S.A.) according to the manufacturer’s protocols. Real-time qPCR was performed in a StepOnePlus Real-Time PCR system (Thermo Fisher, Waltham, MA, U.S.A.) using SYBR green (Thermo Fisher) using Mouse *Smpd1* Real Time PCR Primer Set (Biomol, Hamburg, Germany), Mouse Housekeeping Genes Primer Sets (Biomol), and *ICAM-1* primers according to published sequences [29]. Results were quantified using the 2^−ΔΔCt^ method. Samples were analyzed in triplicates, of which mean values were formed.

### Statistics

First, data distribution was evaluated using Kolmogorov–Smirnov tests, which did not refute normal data distribution. Then, data were analysed by repeated measurement analysis of variance (ANOVA) (comparisons comprising ≥ 2 time-points), oneway ANOVA followed by Tukey’s post hoc tests (comparisons at 1 time-point between ≥ 3 groups) or unpaired two-tailed t tests (comparisons at 1 time-point between 2 groups). Data were presented as means ± standard deviations (SD) [analyses comprising ≥ 2 time-points (that is, LDF measurements) or analyses with small n numbers (that is, Western blots and 3D light sheet microscopy)] or box plots with median (line)/ mean (plus) ± interquartile range (IQR) with minimum and maximum data as whiskers (all other comparisons). Statistical analysis was performed by GraphPad Prism 7.0 software (GraphPad Software, San Diego, CA, U.S.A.). *P* values < 0.05 were considered significant.

## Results

### *Smpd1*^*−/−*^* exacerbates I/R injury in male and female mice*

**Fig. 1:**
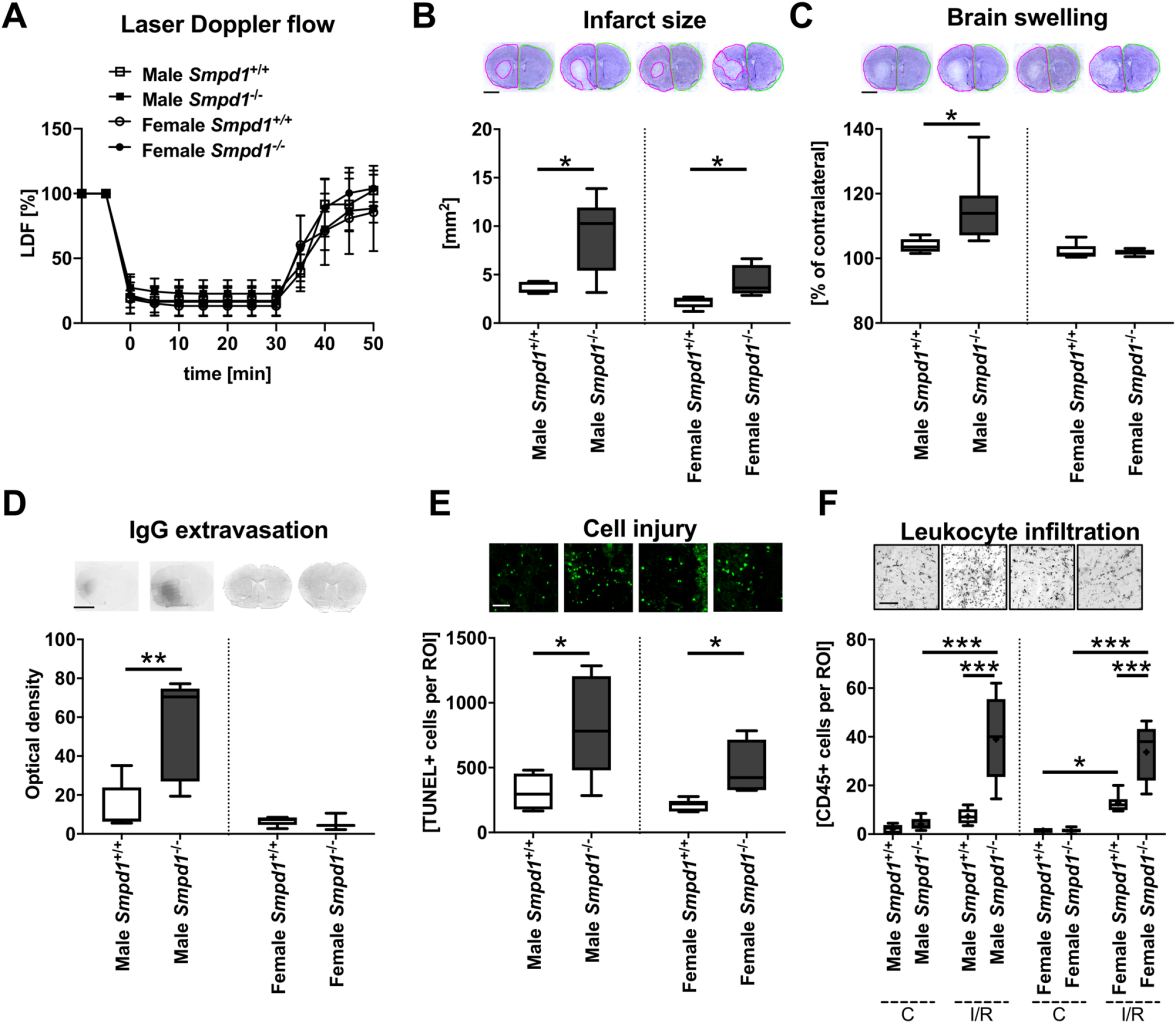
Homozygous *sphingomyelinase phosphodiesterase-1* (*Smpd1*) deficiency exacerbates ischemia/reperfusion (I/R) injury in male and female mice. **a** Laser Doppler flow (LDF) above the core of the vascular territory of the middle cerebral artery, (**b**) infarct size and (**c**) brain swelling evaluated on cresyl violet-stained brain sections, (**d**) serum IgG extravasation in the reperfused ischemic striatum, (**e**) density of DNA-fragmented, that is, irreversibly injured terminal deoxynucleotidyl transferase dUTP nick end labeling (TUNEL) + cells in the reperfused ischemic striatum and (**f**) density of brain infiltrating CD45 + leukocytes in the reperfused ischemic striatum of 12-week-old male or female mice harboring two alleles of the acid sphingomyelinase (Asm) gene *Smpd1* (*Smpd1*^+*/*+^) or no *Smpd1* allele (*Smpd1*^*−/−*^), which were exposed to 30 min of intraluminal middle cerebral artery occlusion (MCAO) followed by animal sacrifice 24 h after reperfusion. Data are means ± SDs (in **a**) or box plots with medians (line)/ means (plus) ± interquartile ranges (IQRs) with minimum and maximum data as whiskers (in **b**-**f**). *p < 0.05, **p < 0.01, ***p < 0.001 (*n* = 5–9 animals per group). Scale bars, 2 mm (in **b**, **c** and **d**)/ 50 µm (in **e** and **f**)

**Fig. 2:**
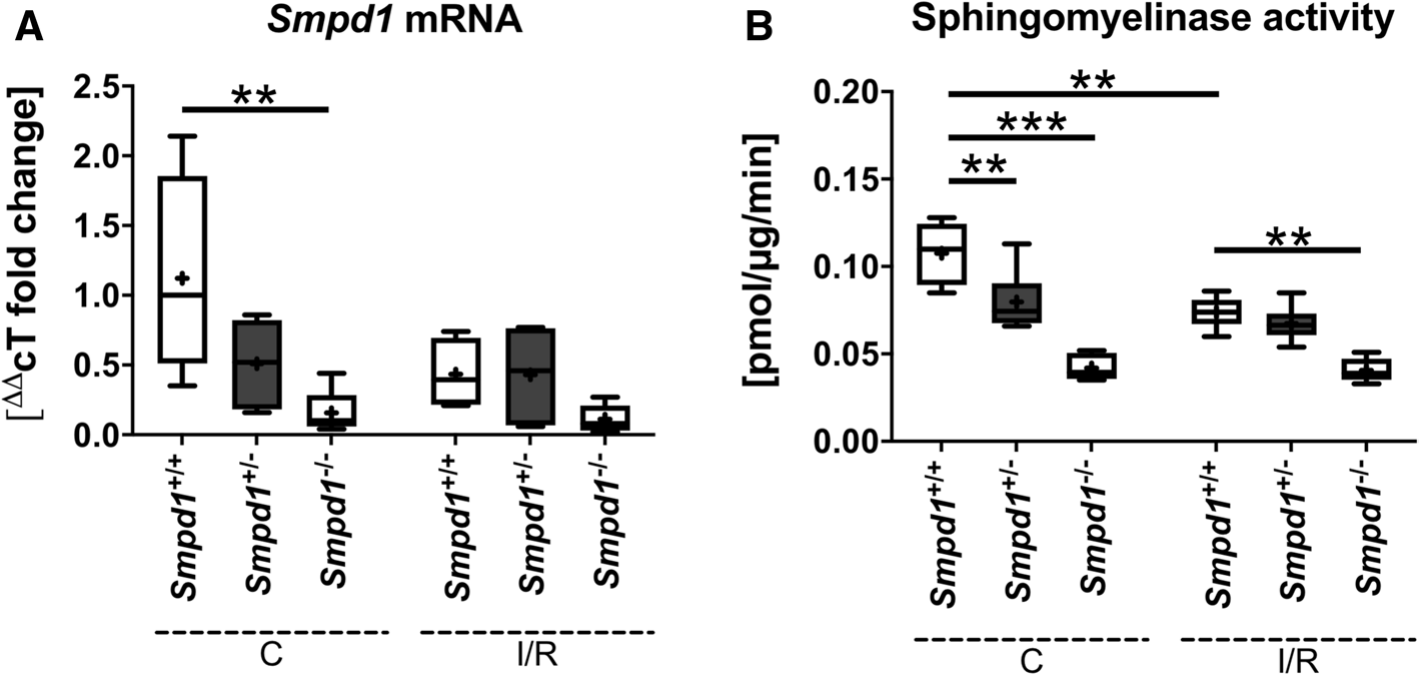
Homozygous *Smpd1* deficiency abrogates, whereas heterozygous *Smpd1* deficiency moderately reduces Asm activity. **a** *Smpd1* mRNA, evaluated by reverse transcriptase quantitative polymerase chain reaction (RT-qPCR), and (**b**) sphingomyelinase activity, evaluated using BODIPY-labeled sphingomyelin as substrate, in contralateral non-ischemic brain tissue and the reperfused ischemic brain tissue of 8-week-old male mice harboring two *Smpd1* alleles (*Smpd1*^+*/*+^), one *Smpd1* allele (*Smpd1*^+/−^) or no *Smpd1* allele (*Smpd1*^*−/−*^), which were exposed to 30 min of MCAO followed by animal sacrifice 24 h after reperfusion. Data are box plots with medians (line)/ means (plus) ± IQRs with minimum and maximum data as whiskers. **p < 0.01, ***p < 0.001 (*n* = 5–6 animals per group independently processed in triplicates)

To study the effect of Asm deficiency on I/R injury, we first exposed 12-week-old male and female *Smpd1*^+*/*+^ and *Smpd1*^*−/−*^ mice to 30 min of intraluminal MCAO, followed by animal sacrifice after 24 h. LDF measurements above the core of the middle cerebral artery territory revealed reproducible ischemia during MCAO in *Smpd1*^+*/*+^ and *Smpd1*^*−/−*^ mice that was followed by the rapid recuperation of blood flow to baseline values after reperfusion onset (Fig. 1a). LDF values did not differ between *Smpd1*^+*/*+^ and *Smpd1*^*−/−*^ mice in both sexes (Fig. 1a). Histochemical studies showed that infarct size was significantly increased in *Smpd1*^*−/−*^ compared with *Smpd1*^+*/*+^ male and female mice (Fig. 1b), as were brain swelling and serum IgG extravasation, a marker of blood–brain barrier permeability, in male mice (Fig. 1c, d). As a consequence of smaller infarcts, female mice exhibited less brain swelling and considerably less IgG extravasation than male mice (Fig. 1c, d). This sex dissociation of blood–brain barrier permeability is noteworthy; it may deserve additional investigation in future studies. In line with the exacerbated brain infarcts, the density of DNA-fragmented, that is, irreversibly injured, TUNEL + cells was significantly increased in the reperfused ischemic striatum of *Smpd1*^*−/−*^ compared with *Smpd1*^+*/*+^ mice (Fig. 1e). Importantly, the brain infiltration of CD45 + leukocytes, which were located in the same areas exhibiting TUNEL + cells, was significantly increased in the reperfused striatum of *Smpd1*^*−/−*^ compared with *Smpd1*^+*/*+^ mice (Fig. 1f). RT-qPCR studies revealed that *Smpd1* mRNA was depleted in brain tissue of *Smpd1*^*−/−*^ mice (Fig. 2a). As a consequence, sphingomyelinase activity was greatly reduced (Fig. 2b). Interestingly, I/R moderately reduced sphingomyelinase activity in brain tissue of *Smpd1*^+*/*+^ mice (Fig. 2b) without affecting *Smpd1* mRNA level (Fig. 2a).

### *Smpd1*^*−/−*^* exacerbates I/R injury independent of mouse age, whereas Smpd1*^+/−^*protects against ischemia*

**Fig. 3:**
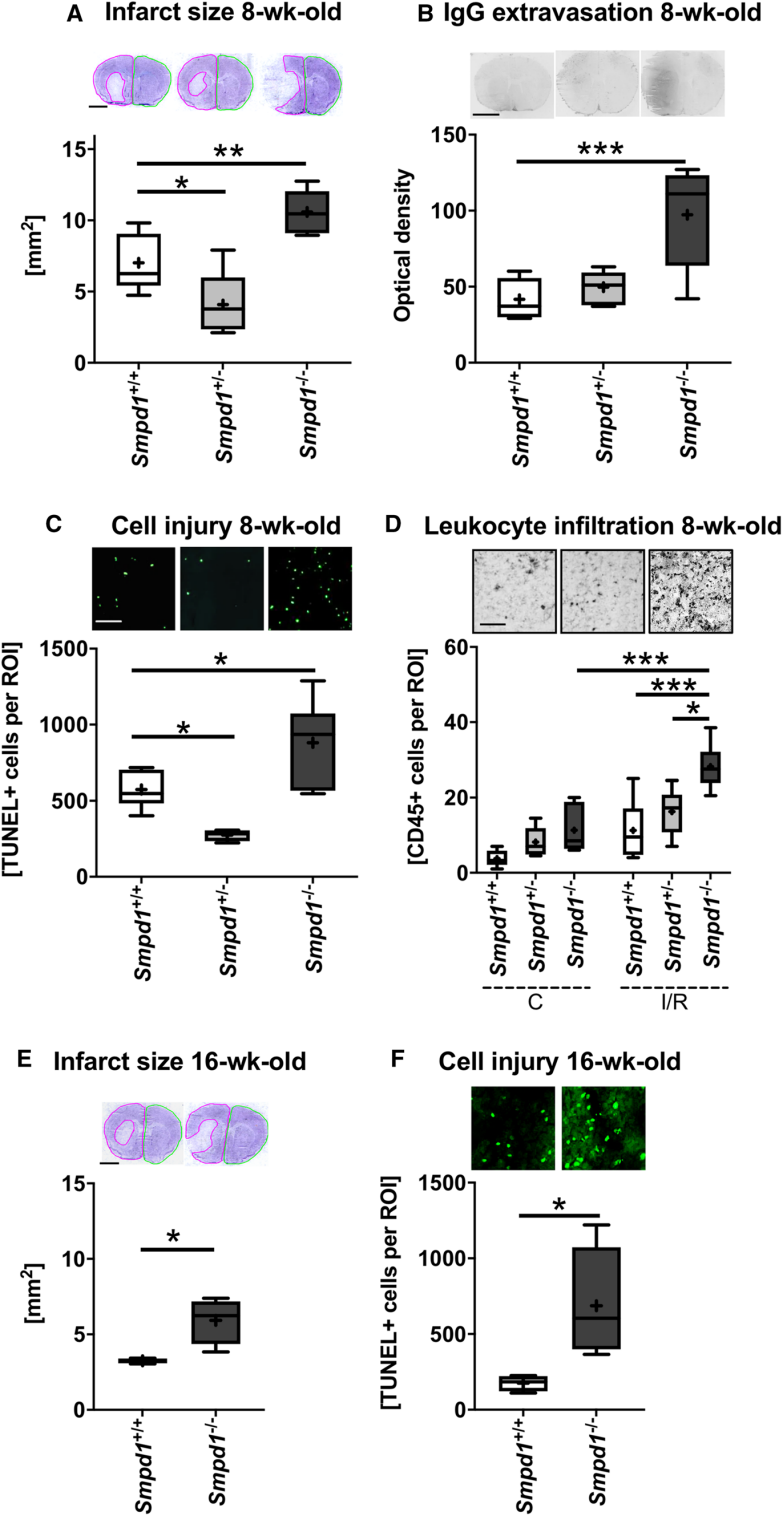
Homozygous *Smpd1* deficiency exacerbates I/R injury independent of animal age, whereas heterozygous *Smpd1* deficiency protects against focal cerebral ischemia. **a** Infarct size evaluated on cresyl violet-stained brain sections, (**b**) serum IgG extravasation in the reperfused ischemic striatum, (**c**) density of DNA-fragmented, that is, irreversibly injured TUNEL + cells in the reperfused ischemic striatum and (**d**) density of brain infiltrating CD45 + leukocytes in the reperfused ischemic striatum of 8-week-old male *Smpd1*^+*/*+^, *Smpd1*^+/−^ or *Smpd1*^*−/−*^ mice, which were exposed to 30 min of MCAO followed by animal sacrifice 24 h after reperfusion. **e** Infarct size evaluated on cresyl violet-stained brain sections and (**f**) density of DNA-fragmented, that is, irreversibly injured TUNEL + cells in the reperfused ischemic striatum of 16-week-old male *Smpd1*^+*/*+^, *Smpd1*^+/−^ or *Smpd1*^*−/−*^ mice, which were exposed to 30 min of MCAO followed by animal sacrifice after 24 h. Together with the data shown in Fig. 1, these results indicate that the exacerbation of I/R injury by *Smpd1*^*−/−*^ is independent of mouse age. Data are box plots with medians (line)/ means (plus) ± IQRs with minimum and maximum data as whiskers. *p < 0.05, **p < 0.01, ***p < 0.001 (*n* = 5–11 animals per group). Scale bars, 2 mm (in **a**, **b** and **e**)/50 µm (in **c**, **d** and **f**)

Since *Smpd1*^*−/−*^ mice develop Niemann-Pick type-A pathology starting at the age of 4–6 months followed by animal death at 8 months [21], we evaluated whether the exacerbation of I/R injury was influenced by the animals’ age. To additionally account for effects of heterozygous *Smpd1* deficiency, we next exposed 8-week-old male *Smpd1*^+*/*+^, *Smpd1*^+/−^ and *Smpd1*^*−/−*^ mice to 30 min intraluminal MCAO. As in the above studies, LDF values did not significantly differ between *Smpd1*^+*/*+^, *Smpd1*^+/−^ and *Smpd1*^*−/−*^ mice (Suppl. Figure 3a). Histochemical studies showed that infarct size, serum IgG extravasation and the density of irreversibly injured TUNEL + cells in the reperfused ischemic striatum (Fig. 3a-c), but not brain swelling (Suppl. Figure 3b) were significantly increased in *Smpd1*^*−/−*^ compared with *Smpd1*^+*/*+^ mice. These parameters were significantly reduced in *Smpd1*^+/−^ compared with *Smpd1*^+*/*+^ mice (Fig. 3a-c) indicating a protection of the heterozygous mice against I/R damage. In view of the novelty of the latter findings, i.e., neuroprotection induced by heterozygous *Smpd1* deficiency and injury exacerbation by homozygous *Smpd1* deficiency, we reevaluated the three genotypes in an independent set of mice, which again showed that *Smpd1*^+/−^ reduced infarct size, whereas *Smpd1*^*−/−*^ increased infarct size and brain swelling (Suppl. Figure 4a, b). The brain infiltration of CD45 + leukocytes was significantly increased in the reperfused striatum of *Smpd1*^−/−^ compared with *Smpd1*^+*/*+^ and *Smpd1*^+/−^ mice (Fig. 3d). Sphingomyelinase activity was moderately reduced in brain tissue of *Smpd1*^+/−^ compared with *Smpd1*^+*/*+^ mice (Fig. 2b).

LDF recordings in 16-week-old male *Smpd1*^+*/*+^ and *Smpd1*^*−/−*^ mice again did not reveal any differences during and after 30 min intraluminal MCAO (Suppl. Figure 3c). Infarct size and the density of irreversibly injured TUNEL + cells in the reperfused striatum (Fig. 3e, f), but not brain swelling (Suppl. Figure 3d) were significantly increased in *Smpd1*^*−/−*^ compared with *Smpd1*^+*/*+^ mice.

**Fig. 4:**
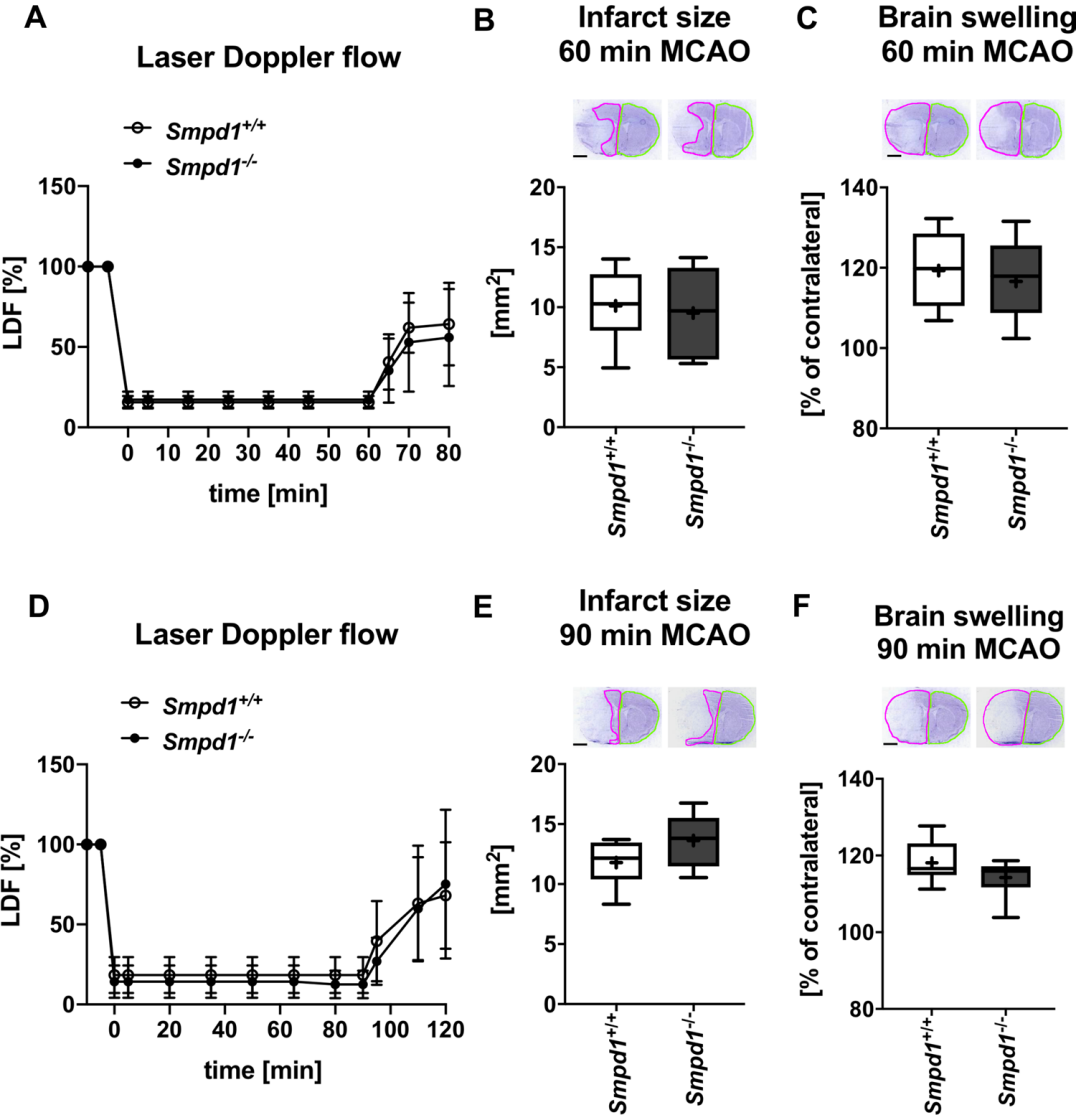
*Smpd1* deficiency did not influence cerebral blood flow, I/R injury or brain swelling in mice exposed to long-lasting 60 or 90 min MCAO. **a**, **d** LDF above the core of the vascular territory of the middle cerebral artery, (**b**, **e**) infarct size and (**c**, **f**) brain swelling evaluated on cresyl violet-stained brain sections of 12-week-old male *Smpd1*^+*/*+^ or *Smpd1*^*−/−*^ mice, which were exposed to 60 min (in **a**-**c**) or 90 min (in **d**-**f**) of MCAO followed by animal sacrifice 24 h after reperfusion. Data are means ± SDs (in **a** and **d**) or box plots with medians (line)/ means (plus) ± IQRs with minimum and maximum data as whiskers (in **b**, **c**, **e** and **f**). No significant differences was found between groups (*n* = 7–8 animals per group). Scale bars, 2 mm (in **b**, **c**, **e** and **f**)

In a sensitivity analysis, we evaluated effects of *Smpd1*^*−/−*^ in mice exposed to 60 min or 90 min MCAO. In both models of longer lasting focal cerebral ischemia, *Smpd1*^*−/−*^ did not influence LDF measurements, infarct size or brain swelling after 24 h (Fig. 4a-f).

### *Smpd1*^*−/−*^* increases ICAM-1 abundance on cerebral microvessels*

**Fig. 5:**
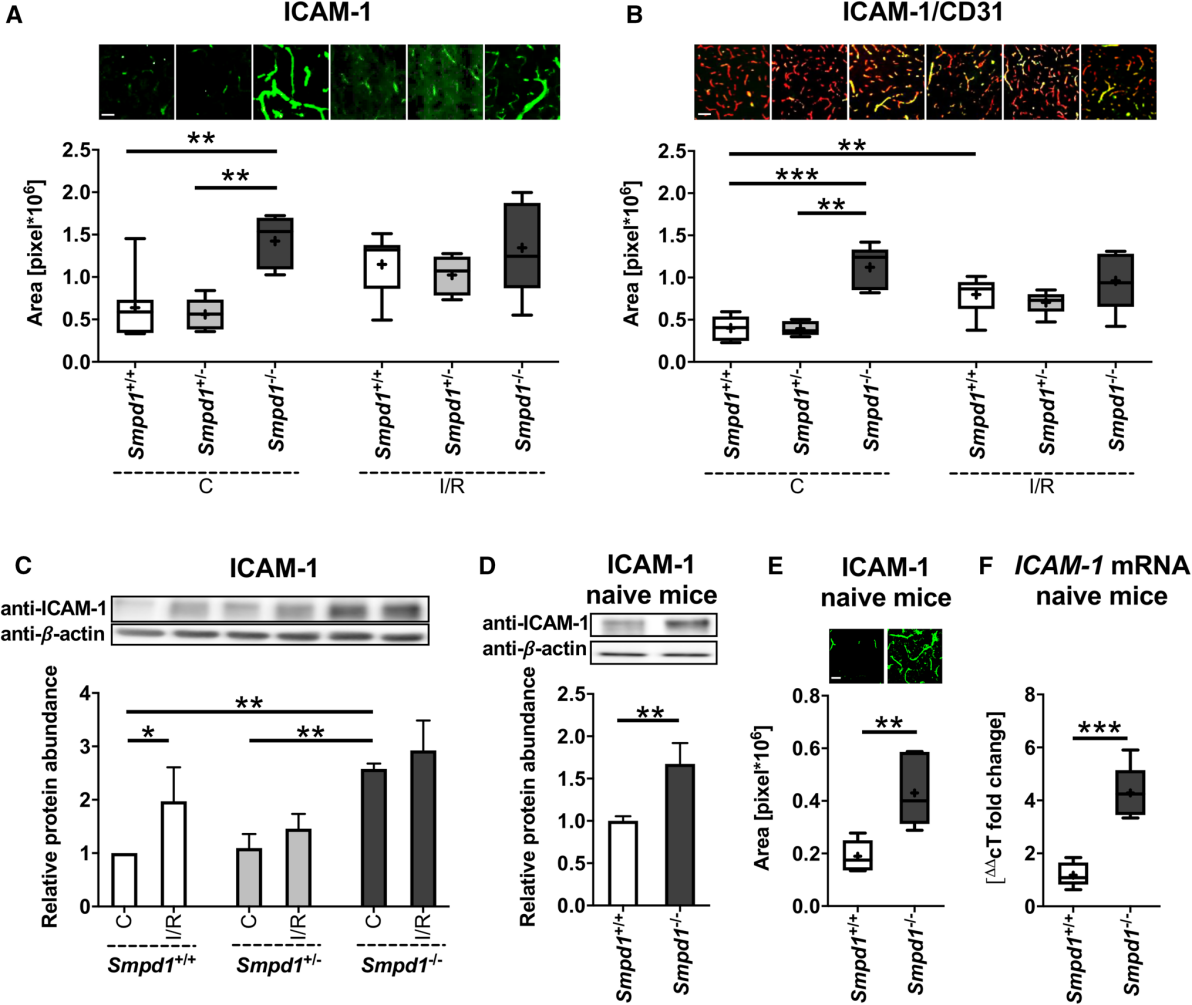
Homozygous, but not heterozygous *Smpd1* deficiency increases ICAM-1 abundance on cerebral microvessels. **a** ICAM-1 + immunofluorescence staining and (**b**) ICAM-1 + / CD31 + immunofluorescence staining on cerebral microvessels in the contralateral non-ischemic striatum and the reperfused ischemic striatum, evaluated by immunohistochemistry, and (**c**) ICAM-1 protein abundance in contralateral non-ischemic striatum and the reperfused ischemic striatum, evaluated by Western blot, of 8-week-old male *Smpd1*^+*/*+^, *Smpd1*^+/−^ or *Smpd1*^*−/−*^ mice, which were exposed to 30 min of MCAO followed by animal sacrifice 24 h after reperfusion. Note that elevated ICAM-1 abundance was similarly evident on cerebral microvessels in the contralateral non-ischemic brain tissue and the reperfused ischemic brain tissue. This prompted us to study (**d**) ICAM-1 + immunofluorescence staining, (**e**) ICAM-1 protein abundance and (**f**) *ICAM-1* mRNA by immunohistochemistry, Western blot and RT-qPCR in corresponding brain areas of 8-week-old male healthy naïve mice which had not been exposed to experimental interventions or anesthesia. Western blots were normalized for β-actin that was used as loading control, and the protein abundance in the *Smpd1*^+*/*+^ group was set as 1. Representative Western blots are also shown. Complete blotting membranes are shown in Suppl. Figure 5. Data are box plots with medians (line)/ means (plus) ± IQRs with minimum and maximum data as whiskers (in **a**, **b**, **e** and **f**) or means ± SDs (in **c** and **d**). *p < 0.05, **p < 0.01, ***p < 0.001 (*n* = 5–8 animals per group (in **a**, **b** and **e**)/ *n* = 3–4 animals per group independently processed in triplicates (in **c** and **d**)/ n = 5–6 animals per group in **f**). Scale bars, 50 µm (in **a**, **b** and **e**)

The brain entry of leukocytes post-I/R is controlled by adhesion molecules such as ICAM-1 [25, 30]. ICAM-1 has previously been shown to cluster in ceramide-rich microdomains on endothelial cells, facilitating actin stress fiber formation and endocytosis [31]. Based on the observation that brain CD45 + leukocyte infiltration was increased by *Smpd1*^*−/−*^, we hypothesized that ICAM-1 abundance was elevated on cerebral microvessels of *Smpd1*^*−/−*^ compared with *Smpd1*^+*/*+^ mice. Immunohistochemistry and Western blot analysis showed that ICAM-1 protein and *ICAM-1* mRNA were indeed increased in the brains of *Smpd1*^*−/−*^ compared with *Smpd1*^+*/*+^ mice (Fig. 5a-f; Suppl. Figure 5). Elevated ICAM-1 abundance on cerebral microvessels was similarly apparent in contralateral non-ischemic and reperfused ischemic tissue (Fig. 5a-c; Suppl. Figure 6), and it was also found on cerebral microvessels of naïve mice not exposed to experimental interventions or anesthesia (Fig. 5d-f).

### *Exacerbated I/R injury in Smpd1*^*−/−*^* mice is mediated by PMNs*

**Fig. 6:**
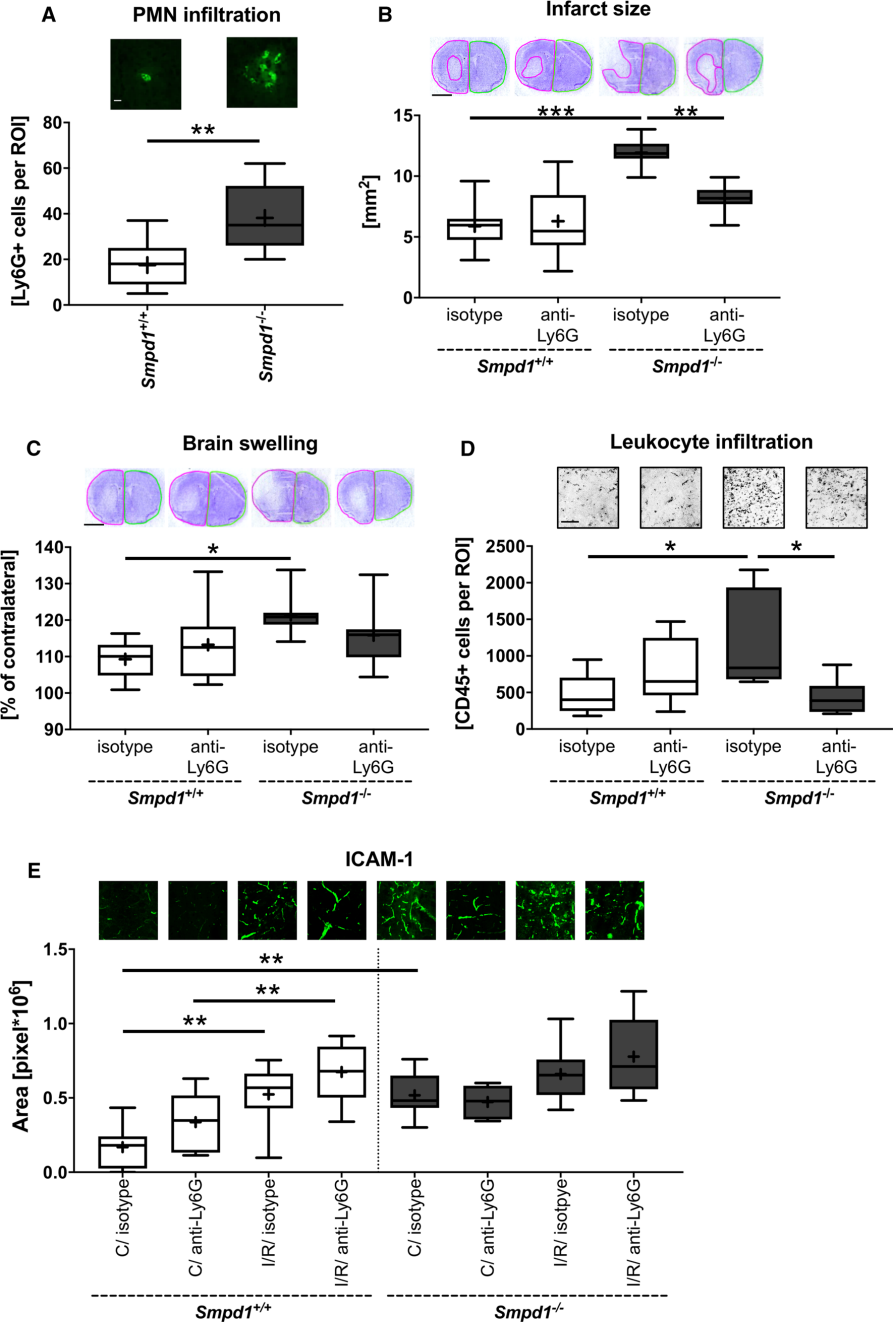
Exacerbated I/R injury in *Smpd1*^*−/−*^ mice is mediated by polymorphonuclear neutrophils (PMNs). **a** Brain-invading Ly6G + PMNs in the ischemic striatum, evaluated by immunohistochemistry, of 8-week-old male *Smpd1*^+*/*+^ or *Smpd1*^*−/−*^ mice, which were exposed to 30 min of MCAO followed by animal sacrifice 72 h after reperfusion. **b** Infarct size and (**c**) brain swelling, evaluated on cresyl violet-stained brain sections, (**d**) brain-infiltrating CD45 + leukocytes in the reperfused ischemic striatum and **e** ICAM-1 immunofluorescence staining in the contralateral non-ischemic striatum and reperfused ischemic striatum, evaluated by immunohistochemistry, of 8-week-old male *Smpd1*^+*/*+^ or *Smpd1*^*−/−*^ mice, which were treated with isotype antibody (as control) or PMN-depleting anti-Ly6G antibody 24 h before and 24 h after 30 min of MCAO followed by animal sacrifice 72 h after reperfusion. Data are box plots with medians (line)/means (plus) ± IQRs with minimum and maximum data as whiskers. *p < 0.05, **p < 0.01, ***p < 0.001 (*n* = 7–10 animals per group). Scale bars, 50 µm (in **a**, **d** and **e**)/ 2 mm (in **b** and **c**)

Polymorphonuclear neutrophils (PMN) have previously been shown to contribute to I/R injury after transient intraluminal MCAO in mice [23, 32]. Considering that PMNs are early invaders of the reperfused ischemic brain, which represent a major percentage of brain leukocyte infiltrates in the first days post-MCAO [33, 34], we asked whether PMNs were increased in reperfused brain tissue by *Smpd1*^*−/−*^. Immunohistochemical studies using the PMN-specific marker Ly6G revealed that a considerable number of brain-infiltrating leukocytes in *Smpd1*^+*/*+^ mice were PMNs and that PMN number was significantly increased by *Smpd1*^−/−^ (Fig. 6a). To test the hypothesis if PMNs were the critical cells that mediated the exacerbated I/R injury, we depleted PMN in *Smpd1*^+/+^ or *Smpd1*^−/−^ mice by delivery of anti-Ly6G antibody as reported previously [23, 32]. LDF recordings above the core of the middle cerebral artery territory did not differ between groups (Suppl. Figure 6). PMN depletion by anti-Ly6G antibody did not influence infarct size, brain swelling or brain CD45 + leukocyte infiltrates in *Smpd1*^+*/*+^ mice exposed to 30 min MCAO followed by 72 h reperfusion, but reversed the exacerbated infarct size and brain swelling (Fig. 6b, c) and reduced CD45 + leukocyte infiltrates by 61 ± 21% (Fig. 6d) in *Smpd1*^−/−^ mice. PMN depletion did not significantly influence ICAM-1 abundance, neither in *Smpd1*^+*/*+^ nor *Smpd1*^*−/−*^ mice (Fig. 6e).

## Discussion

We herein provide evidence that homozygous deficiency of the Asm gene *Smpd1* (*Smpd1*^*−/−*^) exacerbates I/R injury, brain swelling, blood–brain barrier permeability and brain leukocyte and PMN infiltrates independent of the animals’ sex and age in mice exposed to transient intraluminal MCAO, whereas heterozygous *Smpd1*^+/−^ protects against I/R. Intercellular adhesion molecule ICAM-1, which mediates leukocyte and, specifically, PMN entry into the reperfused ischemic brain [25, 30], was increased on cerebral microvessels of *Smpd1*^*−/−*^ compared with *Smpd1*^+*/*+^ and *Smpd1*^+/−^ mice. Using PMN depletion by delivery of anti-Ly6G antibody, we showed that the brain accumulation of PMNs mediates the exacerbated I/R injury in *Smpd1*^*−/−*^ compared with *Smpd1*^+*/*+^ mice. Our results suggest a role of Asm in tempering of leukocyte and, specifically, PMN entry into the reperfused ischemic brain. PMNs have been shown to contribute to I/R injury after transient intraluminal MCAO in the past [23, 32].

That Asm deficiency may predispose to neuronal injury is known from Niemann Pick disease type-A, where progressive neurodegeneration evolves in the cerebral and cerebellar cortex, basal ganglia, brain stem and spinal cord with ataxia, dysarthria and dysphagia starting at the age of 3–4 months as a consequence of a complete loss-of-function *SMPD1* mutation [20]. *Smpd1*^*−/−*^ mice, which have no residual Asm activity, mimic this clinical phenotype. Progressive ataxia is found starting at 4–6 months of age followed by animal death at ~ 8 months [21]. *Smpd1*^+/−^ mice exhibit ~ 50% reduced Asm compared to *Smpd1*^+*/*+^ mice [21]. These mice show no clinical symptoms and develop normally. In the present study, we examined 8-week-old and 12-week-old male and female mice to exclude an interference with Niemann Pick pathology. In a sensitivity analysis, we also assessed 16-week-old mice. Our observations regarding the exacerbation of I/R injury by complete Asm deficiency did not depend on animal age, and they were not influenced by sex. That partial Asm depletion protects the brain against injury has recently been shown in aged mice, in which miRNA-induced *Smpd1* knockdown and tamoxifen-induced endothelial-specific *Smpd1* knockout decreased blood–brain barrier disturbances, neuronal degeneration in cortex and hippocampus and spatial learning deficits [35]. In the aged brain, blood–brain barrier disturbances were found to represent excessive caveolae-mediated transcytosis that was triggered by the overactivation of Asm [35].

After focal cerebral ischemia, reduced I/R injury associated with decreased proinflammatory cytokine production has previously been reported in *Smpd1*^*−/−*^ compared with wildtype mice in a model of 60 min intraluminal MCAO [22]. Similar to the present study, this study examined 2-month-old mice [22]. Different to the present study, sphingomyelinase activity was found to be increased in reperfused brain tissue of *Smpd1*^+*/*+^ mice [22]. The more severe ischemia (60 min vs. 30 min MCAO) very likely explains why they observed elevated sphingomyelinase activity, while we found reduced sphingomyelinase activity in reperfused brain tissue of *Smpd1*^+*/*+^ mice at the time-point of animal sacrifice. The elevated sphingomyelinase activity provides a stringent rationale for why *Smpd1*^*−/−*^ protected against I/R injury in their study but not in our study. To test, if effects of *Smpd1*^*−/−*^ differ depending on the duration of ischemia, we evaluated *Smpd1*^*−/−*^ mice exposed to 60 or 90 min of intraluminal MCAO. In our hands, *Smpd1*^*−/−*^ did not influence I/R injury in both longer lasting MCAO models. A ceiling effect is possible, in which the exacerbation of injury could no more be identified. Hence, 60 min of MCAO may have been too severe to detect neuroprotective effects of *Smpd1*^*−/−*^. Neuroprotective effects of *Smpd1*^*−/−*^ in ischemia durations shorter than 60 min and longer than 30 min cannot be excluded.

Clustering into ceramide-rich membrane platforms, Asm controls immune responses in inflamed tissues. ICAM-1 has been shown to associate with ceramide-rich microdomains on endothelial cells, facilitating actin stress fiber formation and endocytosis [31]. In view of the increased brain leukocyte infiltration, we examined ICAM-1 in the brains of *Smpd1*^+*/*+^, *Smpd1*^+/−^ and *Smpd1*^*−/−*^ mice, showing that ICAM-1 protein and its mRNA were increased on cerebral microvessels upon *Smpd1* deficiency. An increased ICAM-1 abundance associated with increased alveolar leukocyte and, specifically, PMN infiltrates has previously been described in the lungs of *Smpd1*^*−/−*^ mice [36]. In Niemann-Pick type-B pathology, which is characterized by partial Asm deficiency, the elevated ICAM-1 levels have been used for delivering Asm via ICAM-1 targeted polymer nanocarriers [36]. In cultured human cerebral microvascular endothelial cells belonging to the hCMEC/D3 cell line, ICAM-1 abundance was increased, but T cell adhesion and transmigration across hCMEC/D3 cell monolayers were reduced upon lentiviral shRNA-mediated *Smpd1* knockdown [37]. Mechanistically, the phosphorylation of the microvilli protein ezrin was reduced by *Smpd1* knockdown, as was the interaction between the actin filament crosslinking protein filamin and ICAM-1 [37]. To the best of our knowledge, the consequences of *Smpd1* deficiency for the brain invasion of T cells have not yet been assessed. Inhibition of ICAM-1 using a neutralizing antibody has previously been shown to reduce ischemic injury and decrease brain PMN infiltrates after transient MCAO in rats [38].

Probably as a consequence of the elevated ICAM-1 abundance on cerebral microvessels, *Smpd1*^*−/−*^ mice exhibited increased PMN infiltrates in the reperfused ischemic brain. PMN are early invaders of the reperfused brain after intraluminal MCAO [23, 33, 34], which play a pivotal role in I/R injury, since they control the access of other immune cell sets, namely monocytes/ macrophages, T and B cells into the reperfused brain tissue [25]. PMN depletion studies using anti-Ly6G antibody showed that PMNs contribute to I/R injury following transient intraluminal MCAO in normolipidemic mice [23, 25] and hyperlipidemic ApoE^−/−^ mice on Western diet [32]. PMNs abundantly produce and release pro-inflammatory cytokines that promote blood–brain barrier opening, reactive oxygen species that induce structural damage to extracellular matrix proteins, proteases such as elastase and matrix metalloproteinases that degrade extracellular matrix proteins, and DNA traps that promote thrombosis [34, 39]. These effectors likely contribute to I/R injury. By means of PMN depletion by delivery of anti-Ly6G antibody, we indeed found that PMN mediated the exacerbation of I/R injury induced by *Smpd1*^*−/−*^. Of note, only a moderate proportion of brain-invading leukocytes in this study were PMNs (~ 10% based on immunohistochemically determined cell densities). Despite this fact, the brain invasion of total leukocytes was strikingly reduced by PMN depletion by 61 ± 21%, which suggests that, as shown before [25], other immune cell sets, namely monocytes/macrophages, T and B cells, were prevented from entering the brain once PMNs were absent, which may have contributed to brain tissue protection. We have previously shown that anti-Ly6G-mediated PMN depletion selectively eliminates PMNs and not monocytes/ macrophages, T or B cells in peripheral blood of mice exposed to intraluminal MCAO [25]. CD4 and CD8 T cells, although appearing later in the brain than PMNs [39], have previously been shown to contribute to I/R injury after intraluminal MCAO [40]. Our study shows that Asm is indispensable for tempering the brain access of PMNs, which otherwise promote ischemic damage. In this study, PMN depletion by delivery of anti-Ly6G antibody did not reduce I/R injury in *Smpd1*^+*/*+^ mice, which exhibited localized striatal brain infarcts. This observation is in line with a previous study from our group in the same 30 min MCAO model, in which PMN depletion did not protect against I/R injury in normolipidemic mice revealing striatal infarcts but reversed the exacerbated injury in hyperlipidemic mice which had combined corticostriatal stroke [32]. The joint evidence of both studies suggests that the effect of PMN depletion is brain injury severity dependent. It is more significant in combined corticostriatal than pure striatal stroke.

Besides immune-mediated actions, brain parenchymal responses might contribute to the neuroprotective effects of heterozygous *Smpd1* deficiency and injury-aggravating effects of homozygous *Smpd1* deficiency. In a model of glutamate-induced injury of primary mouse and rat oligodendrocytes, reactive oxygen species formation, lipid peroxidation and mitochondrial permeability transition pore opening were reduced by pharmacological Asm inhibition or siRNA-mediated *Smpd1* knockdown, resulting in enhanced oligodendrocyte survival [41]. Both pharmacological Asm inhibition and siRNA-mediated *Smpd1* knockdown induce incomplete Asm deactivation. They resemble heterozygous Asm deficiency in the *Smpd1*^+/−^ mouse. It is tempting to speculate whether peripheral cardiovascular responses may have contributed to the injury effects of heterozygous or homozygous *Smpd1* deficiency. In addition to the brain, ceramide has important functions in the cardiovascular system and, specifically, the heart [42]. In a mouse model of myocardial ischemia/ reperfusion, heterozygous *Smpd1* deficiency did not reduce cardiac injury or improve heart function [43]. In retinal I/R, heterozygous *Smpd1* deficiency protected against retinal degeneration [44]. To the best of our knowledge, no studies hitherto examined effect of homozygous *Smpd1* deficiency on the ischemic heart. A main message of this paper is that Asm and ceramide have injury-limiting roles in addition to their well-known detrimental actions. Tempering excessive Asm overactivation, but maintaining physiological Asm function may be the actual challenge of strategies targeting Asm for stroke recovery.

## Electronic supplementary material

Below is the link to the electronic supplementary material.

Supplementary file1 (PDF 704 kb)

Supplementary file2 (PDF 93 kb)

Supplementary file3 (PDF 764 kb)

Supplementary file4 (PDF 5173 kb)

Supplementary file5 (PDF 162 kb)

Supplementary file6 (PDF 95 kb)

## Data Availability

Not applicable.
