# Supplementary material for: Homozygous Smpd1 deficiency aggravates brain ischemia/ reperfusion injury by mechanisms involving polymorphonuclear neutrophils, whereas heterozygous Smpd1 deficiency protects against mild focal cerebral ischemia
Source: Basic Res Cardiol. 2020 Oct 14;115(6):64. doi: 10.1007/s00395-020-00823-x (PMC7560939; doi:10.1007/s00395-020-00823-x)
Supplement: Supplementary file 1 — Supplementary file1 (PDF 704 kb) [file 395_2020_823_MOESM1_ESM.pdf]

Homozygous *Smpd1* deficiency aggravates brain ischemia/ reperfusion injury by mechanisms involving polymorphonuclear neutrophils, whereas heterozygous *Smpd1* deficiency protects against mild focal cerebral ischemia
Basic research in Cardiology
Nina Hagemann, Ayan Mohamud Yusuf, Carlotta Martiny, Xiaoni Zhang, Christoph Kleinschnitz, Matthias Gunzer, Richard Kolesnick, Erich Gulbins, Dirk M. Hermann
E-Mail: dirk.hermann@uk-essen.de

Supplementary Figure 1

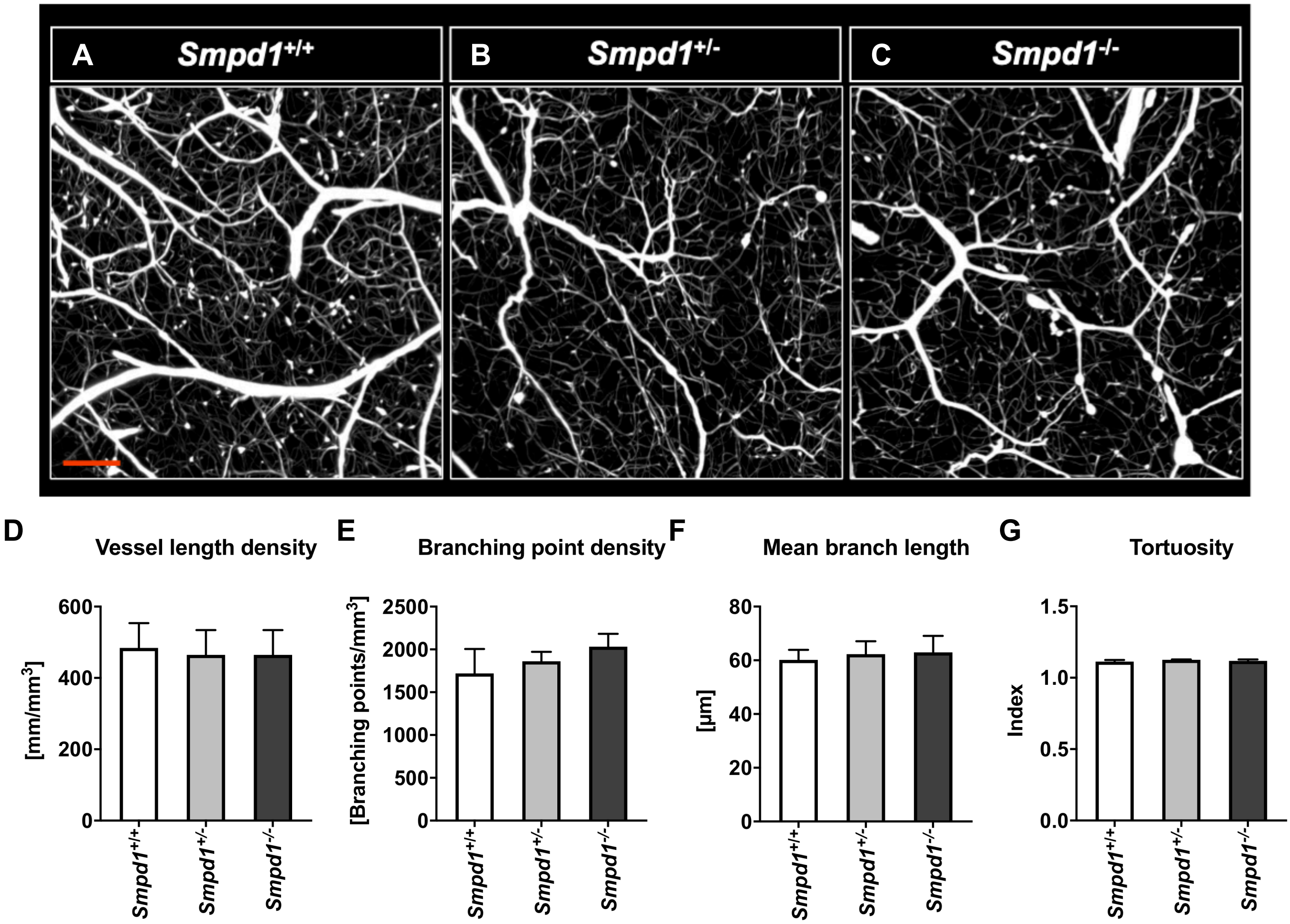

Supplementary Figure 1. Microvascular network characteristics do not differ between mice harboring two alleles of the acid sphingomyelinase (Asm) gene *sphingomyelinase phosphodiesterase-1* (*Smpd1*; *Smpd1*$^{+/+}$), one *Smpd1* allele (*Smpd1*$^{+/-}$) or no *Smpd1* allele (*Smpd1*$^{-/-}$). (A-C) 3D light sheet fluorescent microscopy (LSFM) maximum projection images in the dorsolateral striatum of male *Smpd1*$^{+/+}$, *Smpd1*$^{+/-}$ or *Smpd1*$^{-/-}$ mice, in which microvessels had been labeled with FITC-albumin gelatin. In regions of interest (ROIs) of the striatum measuring 1000 x 500 x 500 µm, **(D)** microvascular length density, **(E)** branching point density, **(F)** mean branch length between two branching points and **(G)** microvascular tortuosity (defined as mean branch length divided by distance between starting-point and endpoint of branches) were evaluated. Data are means ± SD values. No significant differences were noted (n=3-4 animals per group). For details of methods see [24].
