# Supplementary material for: Homozygous Smpd1 deficiency aggravates brain ischemia/ reperfusion injury by mechanisms involving polymorphonuclear neutrophils, whereas heterozygous Smpd1 deficiency protects against mild focal cerebral ischemia
Source: Basic Res Cardiol. 2020 Oct 14;115(6):64. doi: 10.1007/s00395-020-00823-x (PMC7560939; doi:10.1007/s00395-020-00823-x)
Supplement: Supplementary file 2 — Supplementary file2 (PDF 93 kb) [file 395_2020_823_MOESM2_ESM.pdf]

Nina Hagemann, Ayan Mohamud Yusuf, Carlotta Martiny, Xiaoni Zhang, Christoph Kleinschnitz, Matthias Gunzer, Richard Kolesnick, Erich Gulbins, Dirk M. Hermann
E-Mail: dirk.hermann@uk-essen.de

Supplementary Figure 2

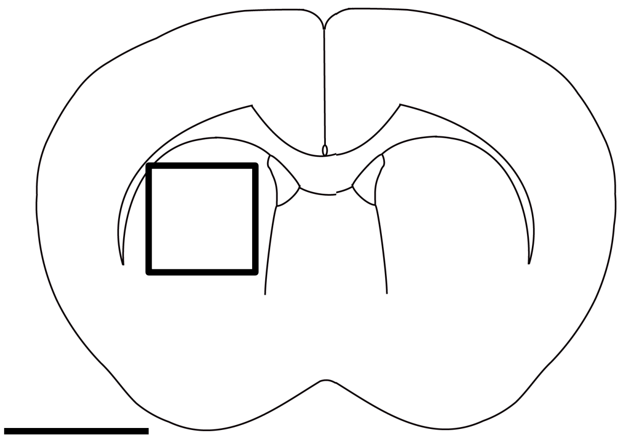

Supplementary Figure 2. Schematic drawing exhibiting brain areas, in which TUNEL stainings and immunohistochemical stainings were analyzed. Note the ROI measuring 1500 x 1500 µm in the dorsolateral striatum, in which cell countings, area analyses and optical density measurements were performed. Scale bar, 2 mm.
