# Supplementary material for: Homozygous Smpd1 deficiency aggravates brain ischemia/ reperfusion injury by mechanisms involving polymorphonuclear neutrophils, whereas heterozygous Smpd1 deficiency protects against mild focal cerebral ischemia
Source: Basic Res Cardiol. 2020 Oct 14;115(6):64. doi: 10.1007/s00395-020-00823-x (PMC7560939; doi:10.1007/s00395-020-00823-x)
Supplement: Supplementary file 3 — Supplementary file3 (PDF 764 kb) [file 395_2020_823_MOESM3_ESM.pdf]

Nina Hagemann, Ayan Mohamud Yusuf, Carlotta Martiny, Xiaoni Zhang, Christoph Kleinschnitz, Matthias Gunzer, Richard Kolesnick, Erich Gulbins, Dirk M. Hermann
E-Mail: dirk.hermann@uk-essen.de

Supplementary Figure 3

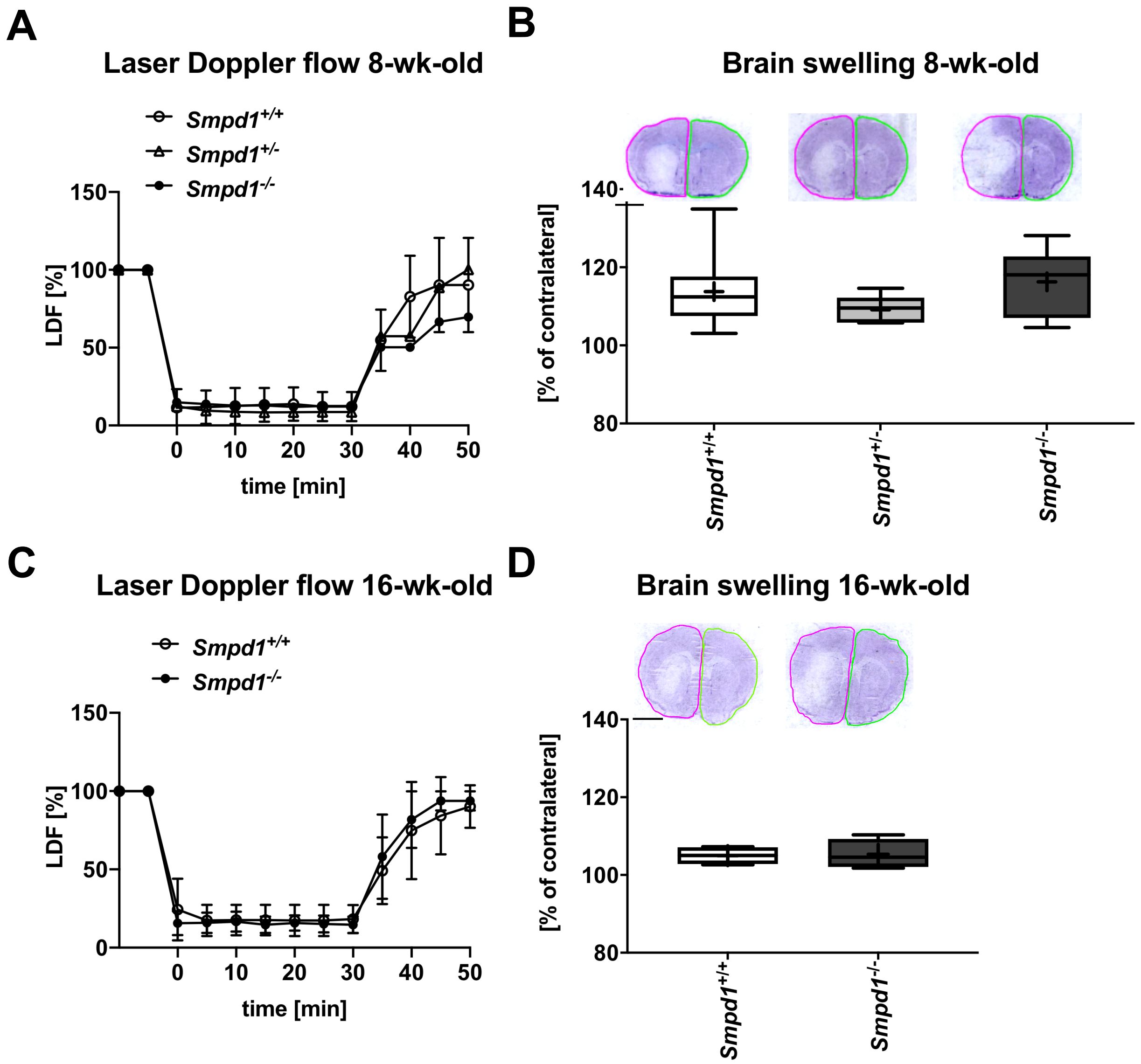

Supplementary Figure 3. *Smpd1* deficiency did not influence cerebral blood flow or brain swelling in 8-week-old or 16-week-old mice exposed to transient middle cerebral artery occlusion (MCAO). (A, C) Laser Doppler flow (LDF) above the core of the vascular territory of the middle cerebral artery, and (B, D) brain swelling evaluated on cresyl violet-stained brain sections of 8-week-old (in A and B) or 16-week-old (in C and D) male mice harboring two alleles of *Smpd1* (*Smpd1*$^{+/+}$), one Smpd1 allele (*Smpd1*$^{+/-}$) or no *Smpd1* allele (*Smpd1*$^{-/-}$), which were exposed to 30 minutes of MCAO followed by animal sacrifice 24 hours after reperfusion. Data are means ± standard deviations (SDs) (in A and C) or box plots with medians (line)/ means (plus) ± interquartile ranges (IQRs) with minimum and maximum data as whiskers (in B and D). No significant differences were found between groups (n=4-11 animals per group). Scale bars, 2 mm (in B and D).
