# Supplementary material for: Homozygous Smpd1 deficiency aggravates brain ischemia/ reperfusion injury by mechanisms involving polymorphonuclear neutrophils, whereas heterozygous Smpd1 deficiency protects against mild focal cerebral ischemia
Source: Basic Res Cardiol. 2020 Oct 14;115(6):64. doi: 10.1007/s00395-020-00823-x (PMC7560939; doi:10.1007/s00395-020-00823-x)
Supplement: Supplementary file 4 — Supplementary file4 (PDF 5173 kb) [file 395_2020_823_MOESM4_ESM.pdf]

Homozygous *Smpd1* deficiency aggravates brain ischemia/ reperfusion injury by mechanisms involving polymorphonuclear neutrophils, whereas heterozygous *Smpd1* deficiency protects against mild focal cerebral ischemia
Basic research in Cardiology
Nina Hagemann, Ayan Mohamud Yusuf, Carlotta Martiny, Xiaoni Zhang, Christoph Kleinschnitz, Matthias Gunzer, Richard Kolesnick, Erich Gulbins, Dirk M. Hermann
E-Mail: dirk.hermann@uk-essen.de

Supplementary Figure 4

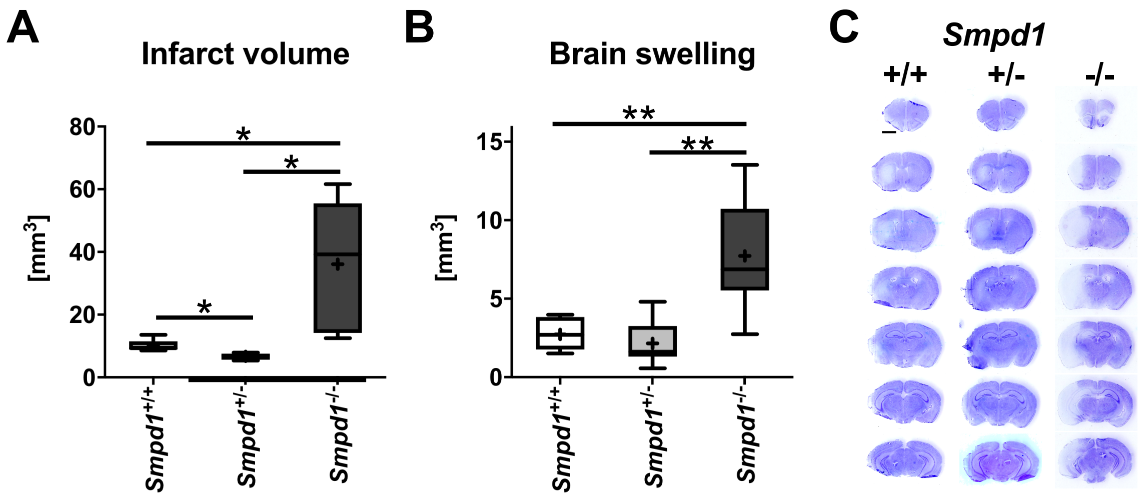

Supplementary Figure 4. Homozygous *Smpd1* deficiency exacerbates I/R injury independent of animal age, whereas heterozygous *Smpd1* deficiency protects against focal cerebral ischemia. (A) Infarct volume and **(B)** brain swelling evaluated on cresyl violet-stained brain sections of 12-week-old male $Smpd1^{+/+}$, $Smpd1^{+/-}$ or $Smpd1^{-/-}$ mice, which were exposed to 30 minutes of MCAO followed by animal sacrifice 24 hours after reperfusion. Representative cresyl violet-stained brain sections are shown in **(C)**. This data set confirms the observations of **Figure 3** in an independent cohort of mice. Data were analyzed by oneway ANOVA, followed by Bonferroni-corrected posthoc t-tests. Data are box plots with medians (line)/ means (plus) ± IQRs with minimum and maximum data as whiskers. *p<0.05, **p<0.01 (n=6-10 animals per group). Scale bar, 2 mm.
