# Supplementary material for: Homozygous Smpd1 deficiency aggravates brain ischemia/ reperfusion injury by mechanisms involving polymorphonuclear neutrophils, whereas heterozygous Smpd1 deficiency protects against mild focal cerebral ischemia
Source: Basic Res Cardiol. 2020 Oct 14;115(6):64. doi: 10.1007/s00395-020-00823-x (PMC7560939; doi:10.1007/s00395-020-00823-x)
Supplement: Supplementary file 5 — Supplementary file5 (PDF 162 kb) [file 395_2020_823_MOESM5_ESM.pdf]

Homozygous *Smpd1* deficiency aggravates brain ischemia/ reperfusion injury by mechanisms involving polymorphonuclear neutrophils, whereas heterozygous *Smpd1* deficiency protects against mild focal cerebral ischemia

Basic research in Cardiology

Nina Hagemann, Ayan Mohamud Yusuf, Carlotta Martiny, Xiaoni Zhang, Christoph Kleinschnitz, Matthias Gunzer, Richard Kolesnick, Erich Gulbins, Dirk M. Hermann
E-Mail: dirk.hermann@uk-essen.de

Supplementary Figure 5

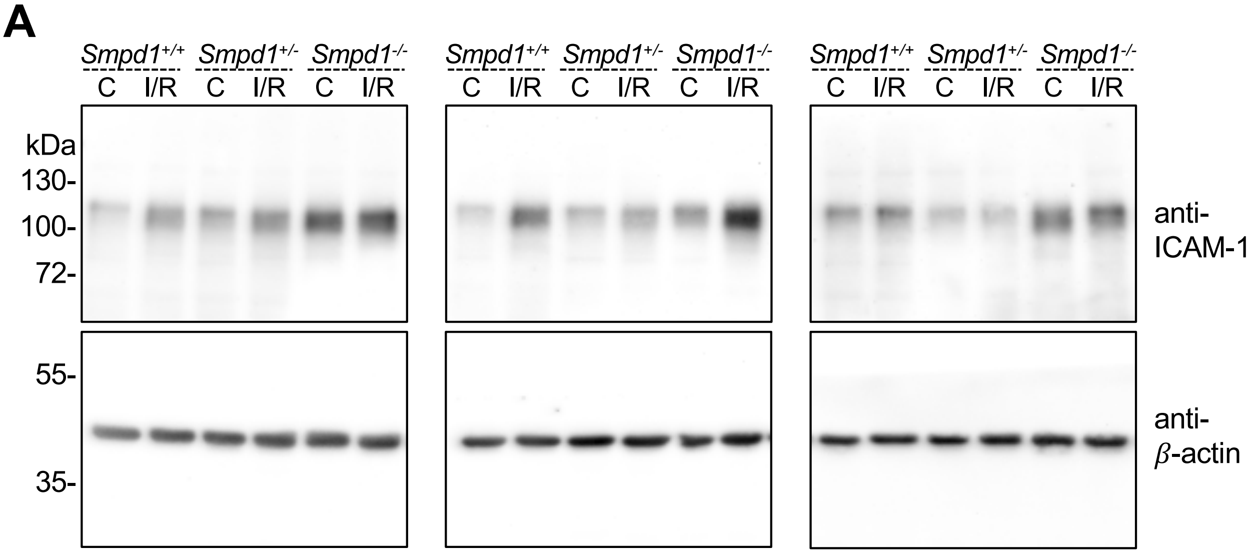

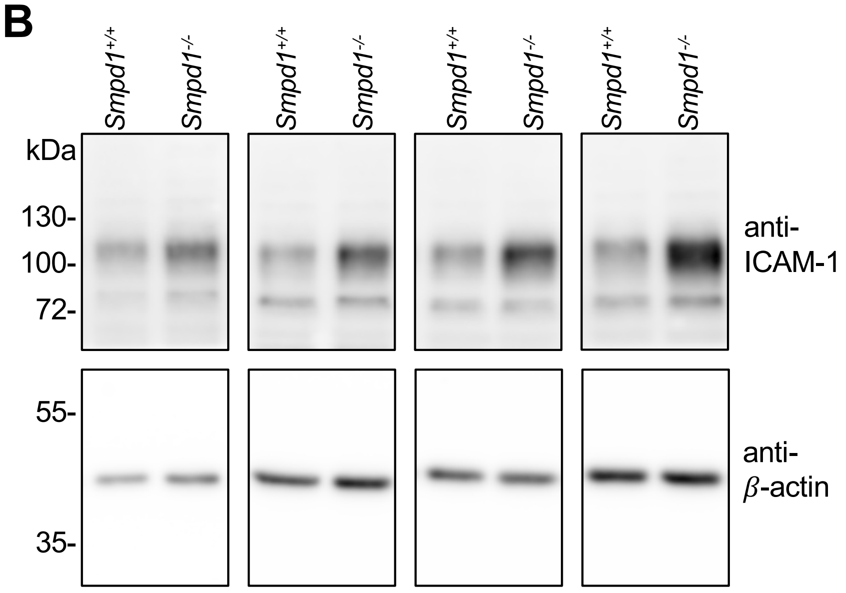

Supplementary Figure 5. Homozygous, but not heterozygous *Smpd1* deficiency increases ICAM-1 abundance on cerebral microvessels. Complete Western blot membranes exhibiting ICAM-1 protein abundance **(A)** in the contralateral non-ischemic striatum (C) and the reperfused ischemic striatum (I/R) of 8-week-old male *Smpd1*$^{+/+}$, *Smpd1*$^{+/-}$ or *Smpd1*$^{-/-}$ mice, which were exposed to 30 minutes of MCAO followed by animal sacrifice 24 hours after reperfusion and **(B)** in the striatum of 8-week-old male naïve *Smpd1*$^{+/+}$, *Smpd1*$^{+/-}$ or *Smpd1*$^{-/-}$ mice which had not been exposed to experimental interventions or anesthesia. β-actin blots, which were used as loading controls, are also shown. For the semiquantitative analysis of Western blots see **Figure 5** of the main manuscript.
