# Supplementary material for: Homozygous Smpd1 deficiency aggravates brain ischemia/ reperfusion injury by mechanisms involving polymorphonuclear neutrophils, whereas heterozygous Smpd1 deficiency protects against mild focal cerebral ischemia
Source: Basic Res Cardiol. 2020 Oct 14;115(6):64. doi: 10.1007/s00395-020-00823-x (PMC7560939; doi:10.1007/s00395-020-00823-x)
Supplement: Supplementary file 6 — Supplementary file6 (PDF 95 kb) [file 395_2020_823_MOESM6_ESM.pdf]

Homozygous *Smpd1* deficiency aggravates brain ischemia/ reperfusion injury by mechanisms involving polymorphonuclear neutrophils, whereas heterozygous *Smpd1* deficiency protects against mild focal cerebral ischemia
Basic research in Cardiology
Nina Hagemann, Ayan Mohamud Yusuf, Carlotta Martiny, Xiaoni Zhang, Christoph Kleinschnitz, Matthias Gunzer, Richard Kolesnick, Erich Gulbins, Dirk M. Hermann
E-Mail: dirk.hermann@uk-essen.de

Supplementary Figure 6

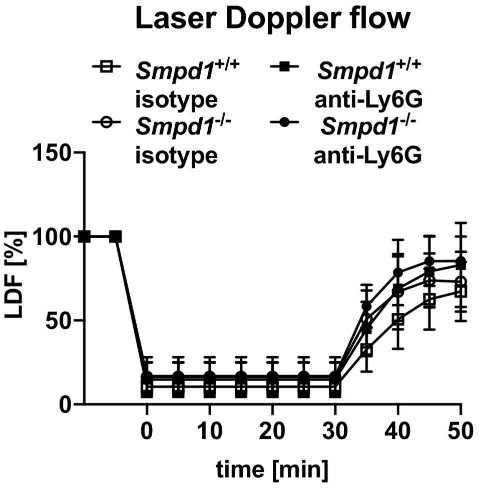

Supplementary Figure 6. PMN depletion does not influence cerebral laser Doppler flow (LDF). LDF above the core of the vascular territory of the middle cerebral artery of 8-week-old male *Smpd1*$^{+/+}$ or *Smpd1*$^{-/-}$ mice, which were treated with isotype antibody (as control) or PMN-depleting anti-Ly6G antibody 24 hours before and 24 hours after 30 minutes of MCAO followed by animal sacrifice 72 hours after reperfusion. Data are means ± SDs. No significant differences were noted between groups (n=7-10 animals per group).
